# Level of prematurity as a predictor for the activity of retinopathy of prematurity and strabismus

**DOI:** 10.22336/rjo.2023.10

**Published:** 2023

**Authors:** Aida Pidro Gadzo, Ajla Pidro Miokovic, Jasmina Alajbegovic Halimic, Denisa Zvizdic

**Affiliations:** * Ophthalmology Department, “Prim. Dr. Abdulah Nakaš” General Hospital, Sarajevo, Bosnia and Herzegovina; ** Polyclinic Vukas, Zagreb, Croatia; *** “Dr. Halimic” Private Ophthalmological Office, Sarajevo, Bosnia and Herzegovina; **** Clinic for Eye Disease, Clinical Centre University of Sarajevo, Sarajevo, Bosnia and Herzegovina

**Keywords:** ROP, birth weight, gestation age

## Abstract

**Objective:** To evaluate the prevalence of strabismus in premature children after the screening for retinopathy of prematurity (ROP) and to determine whether the level of prematurity (birth weight and gestation age) can be used as a predictor for both strabismus development and disease activity.

**Methods:** This is a retrospective study, conducted in the Clinic for Eye Disease, section for Pediatric Ophthalmology of the Clinical Centre University of Sarajevo during the period from December 2013 until January 2017. 126 patients were involved. The inclusion criteria were gestational age ≤ 34 weeks, birthweight ≤ 2000 g and performed screening test for ROP. Patients were divided into three groups: patients without ROP, patients with spontaneous regression and patients with active ROP.

**Results:** 52.4% patients were suffering from strabismus (30.2% esotropia, 22.2% exotropia). All the patients with active ROP had esotropia (60.0%). The average birth weight and gestational age were lower in patients with strabismus (1371.3 ± 58.0 g and 29.7 ± 0.4 weeks respectively).

**Conclusion:** Lower gestational age and birth weight of premature children showed the increased probability of strabismus development especially esotropia. Prematurity level was significantly lower in patients with active ROP compared to the rest of the patients.

**Abbreviations:** ROP = Retinopathy of prematurity, ICROP = International Classification of Retinopathy of prematurity, BCVA = best corrected visual acuity

## Introduction

The development of Neonatal care units worldwide has increased a survival rate in prematurely born children. Premature birth leads to different systemic sequalae, including ophthalmological ones. The most important one is the retinopathy of prematurity (ROP), which can then increase the prevalence of other ophthalmological conditions, including refractive anomalies, strabismus, visual impairment, amblyopia, color vision impairment and visual field defects and nystagmus as the most frequent [**1**]. 

As the incidence of ROP has increased, it became one of the most investigated topics around the world [**2**]. There are different risk factors for the development of ROP, among which gestation age, birth weight and oxygen therapy are the most important [**3**]. Other, less investigated, factors include anemia, apnea, mechanical ventilation, surfactant therapy, etc. [**4**].

The aim of this research was to evaluate the prevalence of the active form of ROP and strabismus in prematurely born children. We also aimed to determine whether the level of prematurity (birth weight and gestation age) could be used as a predictor for both strabismus development and disease activity.

## Methods

This is a clinical, descriptive, retrospective study, conducted in the Clinic for Eye Disease, section for Pediatric Ophthalmology of the Clinical Centre University of Sarajevo. The total number of patients was 126. All the patients were prematurely born and were screened for ROP during the period from December 2013 until January 2017. They also had regular follow-ups during the one-year period after screening. The screening was performed following German guidelines, starting 6 weeks after birth and by performing regular follow-ups until either full retinal vascularization or disease regression [**5**]. The guidelines of International classification of retinopathy of prematurity (ICROP) were used for the determination of the follow-up’s frequency [**6**].

Inclusion criteria for the research were birth weight ≤ 2000, gestation age ≤ 34 weeks and infants with other risk factors recognized by neonatologists. Exclusion criteria were irregular follow-ups, congenital anomalies and full-term infants.

Therefore, patients were divided into the three groups: group A - prematurely born infants without any signs of ROP, group B - prematurely born infants who a had less severe form of ROP, which spontaneously regressed during time, and group C - prematurely born infants who had an active form of ROP and were treated according to the disease severity.

Data were obtained from the patients’ medical history. It contained demographic data, as well as gestation age, birth weight and medical report from the first examination and all further follow ups. The report contained the data of standard pediatric ophthalmological examination, including best-corrected visual acuity (BCVA) assessment using Lea symbols, cycloplegic refraction and keratometry with the calculation of spherical equivalent, orthoptic examination for strabismus diagnosis and fundus examination using indirect ophthalmoscopy with the evaluation of ROP stage. Patients were also classified according to the deviation pattern from the primary position gaze into orthophoria and heterotropia (esotropia and exotropia). 

The study was performed in the accordance with the Declaration of Helsinki and was approved and supported by the Ethical Committee of the Clinical Center University of Sarajevo. Written informed consent was obtained from each participant. Patient records/ information was anonymized and de-identified prior to analysis. Gathered data were presented through tables and graphs using percentage, mean value, standard deviation, standard error, maximum and minimum values. χ2 test and Spearman correlation test were used and the results were considered statistically significant if p<0.05. The analysis was performed using the statistical package IBM Statistics SPSS v 23.0, and Microsoft Word and Excel (2010).

## Results

126 prematurely born infants were divided into three groups. Group A had 15 patients (11.9%), group B 106 patients (84.1%) and group C had 5 patients (4.0%). 

Strabismus was diagnosed in 66 patients (52.4%), of whom 38 patients (30.2%) had esotropia and 28 (22.2%) had exotropia (**Table 1**) (rho=-0,124, p=0,090). Group A had 6 patients (40.0%) with strabismus, among whom esotropia and exotropia were equally distributed (20.0% each). Group B was the leading group with strabismus diagnosis made up of 57 patients in total (53.8%), with esotropia (30.2%) more frequent than exotropia (23.6%). Group C had the highest percentage of patients with strabismus (60.0%), with all the patients diagnosed with esotropia. 

**Table 1:** The correlation between ROP activity and strabismus

			Groups according to ROP severity			Total
			Group C	Group B	Group A	
Strabismus	Orthophoria	N	2	49	9	60
		%	40.0	46.2	60.0	47.6
	Exotropia	N	0	25	3	28
		%	0.0	23.6	20.0	22.2
	Esotropia	N	3	32	3	38
		%	60.0	30.2	20.0	30.2
Total		N	5	106	15	126
		%	100.0	100.0	100.0	100.0
χ2=3.810; p=0.432, rho=-0.124; p=0.090 *ROP = retinopathy of prematurity						

**Fig. 1:**
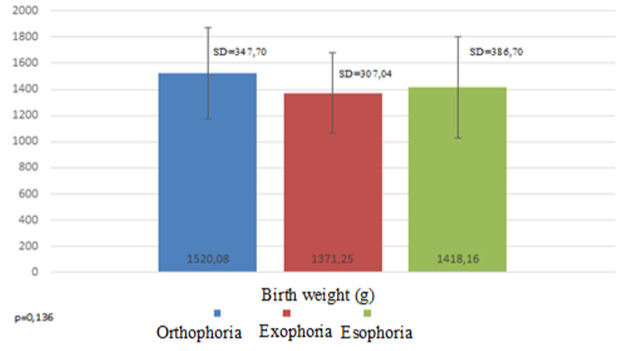
Strabismus prevalence according to the birth weight

The average birth weight in this research was 1456.3 g (min 640.0, max 2000.0) with most patients being in the range of 1200.0 and 1700.0 g. The highest average birthweight was in patients with orthophoria 1520.1 ± 44.9 g (min. 860.0, max. 2000.0) compared to the patients with heterotropia. Esotropia had a slightly higher average birth weight (1418.2 ± 62.7 g) than exotropia (1371.3 ± 58.0 g). Esotropia had lower minimum birth weight (640.0 g) compared to 820.0 g in exotropia (**Fig. 1**).

**Fig. 2:**
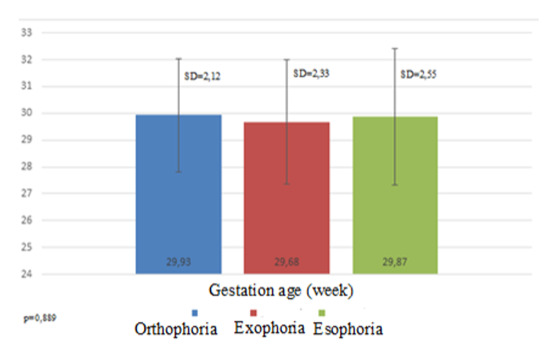
Strabismus prevalence according to the gestation age

The average gestation age in this research was 29.6 weeks (min. 24, max. 34), with the most patients in the range of 28 to 31 weeks’ gestation. There was a small difference in the average gestation age in children with orthophoria (29.9 ± 0.3 weeks) and heterotropia (exotropia 29.7 ± 0.4 and esotropia 29.9 ± 0.4) (**Fig. 2**).

## Discussion

Our results showed that most prematurely born infants developed some stage of ROP, but most of them spontaneously regressed and did not need treatment. The presence of an acute disease is a lot lower compared to other older studies and can be explained by the development of Pediatrics and Neonatology units in the last two decades, which provided an increased survival rate of children with low gestation age and birth weight [**1**].

Strabismus diagnosis was determined during the period of follow-ups since it is sometimes hard to make a diagnosis in children younger than six weeks due to the decrease in coordination of the ocular movement. It is of utmost importance to recognize strabismus and to start the treatment in the early age with either conservative or surgical method to provide a proper visual development and prevent amblyopia. Our study also showed that the prevalence of strabismus was higher in children who were prematurely born. The study of Fieß et al. [**7**] demonstrated a lower prevalence of strabismus than our study (23%). A higher prevalence was also reported by VanderVeen et al. (42.2%) [**8**]. The difference can be explained by the different inclusion and exclusion criteria, different time of follow-up, higher number of patients with an active form of the disease and different study designs. However, when compared to strabismus prevalence in full-term children (0.7-9.9%), all results from similar studies showed a significantly higher prevalence of strabismus in prematurely born children [**9**]. 

Overall, esotropia was more frequent in our study compared to exotropia in these children, especially in the active form of retinopathy of prematurity, in which all patients developed esotropia. Fieß et al. have also reported esotropia as a more frequent type of strabismus [**7**]. Their study also showed the correlation between strabismus and hyperopia, with esotropia being more related to it than exotropia. Many other studies also confirm our findings [**10**]. Even though the topic has not been fully investigated yet, some studies explain strabismus development in a connection to the presence of active ROP, increased incidence of refractive anomalies and neurological conditions, such as brain injuries, which are more frequent in premature children [**11**-**13**].

VanderVeen et al. reported that most children who were diagnosed with strabismus at the time of examination remained with this diagnosis, but some children managed to stabilize the alignment, even without the surgery [**8**].

There are several studies with the same objectives as in our study and some of them reported different results. Cotter et al. [**14**] reported a higher prevalence in strabismus in patients with low gestational age, while Gulati et al. [**15**] reported the higher prevalence in patients with low birth weight and no association with gestation age. The average birth weight in our study was the highest in patients with orthophoria. The lowest was in exophoria, even though esophoria had the lowest minimum birth weight. The average gestation age showed the same trend as birth weight, but with a very small difference. Our results are consistent with other studies that indicate birth weight as a more significant predictive factor for strabismus [**15**,**16**]. Fieß et al. reported a strong association with strabismus of both gestation age and birth weight [**7**]. This means that as the infant is born early and has a lower gestation age, there is a higher risk of developing one of the ocular complications, such as strabismus. 

The most significant results of this study were the impact of low gestation age and birth weight on disease activity. Patients with the lowest gestation age and birth weight had an increased chance of having the active form of the disease, which required a prompt treatment. The treatment of ROP included laser photocoagulation, intravitreal anti-VEGF therapy and vitrectomy. Anti-VEGF therapy used at the time was bevacizumab. Thus, it is important to recognize the main risk factors and to perform more frequent follow-ups in these children.

The strength of this study is that there are no studies in Bosnia and Herzegovina that have investigated gestation age and birth weight as a predictive factor for the development of the active form of ROP and, overall, there is a small number of studies investigating the development of strabismus in these patients. The limitation of this study is a low number of patients, especially in the group with the active form of the disease. The study was conducted in one Clinical Center and can be improved by uniting the data from one more Clinical Center in Bosnia and Herzegovina that treated ROP. One more improvement could be made by comparing both refractive errors and strabismus with these variables. A control group of the same number of full-term children investigated in a prospective manner could increase the significance of this whole research.

## Conclusion

Strabismus has the higher prevalence in prematurely born children. This study provided proof that there is a correlation of strabismus with the level of prematurity and that both gestation age and birth weight could be used as a predictor for both the development of the active form of the disease and the strabismus diagnosis, especially esotropia. If diagnosed on time, the treatment for strabismus and amblyopia prevention could be performed when needed and therefore could provide a better vision and a better quality of life. 

**Conflicts of Interest statement**

The authors declare that there are no conflicts of interest. 

**Informed Consent and Human and Animal Rights statement**

Informed consent has been obtained from the patient included in this study. 

**Authorization for the use of human subject**

Ethical approval: The case report related to human use complies with all the relevant national regulations, institutional policies, is in accordance with the tenets of the Helsinki Declaration, and has been approved by the review board of the Veneto Eye Bank Foundation, Venice, Italy. 

**Acknowledgments**

None. 

**Sources of Funding**

None.

**Financial Disclosure(s)**

None.
